# Estrogen receptor positive breast cancers and their association with environmental factors

**DOI:** 10.1186/1476-072X-10-32

**Published:** 2011-05-10

**Authors:** Sophie St-Hilaire, Rakesh Mandal, Amy Commendador, Sylvio Mannel, DeWayne Derryberry

**Affiliations:** 1 Department of Biological Sciences, 650 Memorial Drive, Idaho State University, Pocatello, ID 83209, 208-282-5416, USA; 2 Waisman Center, University of Wisconsin, Madison, WI 53705, USA; 3 Department of Geosciences, Idaho State University, Pocatello, ID 83209, USA; 4 Department of Environmental Studies, Cottey College, 1000 W. Austin, Nevada, MO 64772, USA; 5 Mathematics Department, Idaho State University, Pocatello, ID 83209, USA

## Abstract

**Background:**

Epidemiological studies to assess risk factors for breast cancer often do not differentiate between different types of breast cancers. We applied a general linear model to determine whether data from the Surveillance, Epidemiology, and End Results Program on annual county level age-adjusted incidence rates of breast cancer with and without estrogen receptors (ER+ and ER-) were associated with environmental pollutants.

**Results:**

Our final model explained approximately 38% of the variation in the rate of ER+ breast cancer. In contrast, we were only able to explain 14% of the variation in the rate of ER- breast cancer with the same set of environmental variables. Only ER+ breast cancers were positively associated with the EPA's estimated risk of cancer based on toxic air emissions and the proportion of agricultural land in a county. Meteorological variables, including short wave radiation, temperature, precipitation, and water vapor pressure, were also significantly associated with the rate of ER+ breast cancer, after controlling for age, race, premature mortality from heart disease, and unemployment rate.

**Conclusions:**

Our findings were consistent with what we expected, given the fact that many of the commonly used pesticides and air pollutants included in the EPA cancer risk score are classified as endocrine disruptors and ER+ breast cancers respond more strongly to estrogen than ER- breast cancers. The findings of this study suggest that ER+ and ER- breast cancers have different risk factors, which should be taken into consideration in future studies that seek to understand environmental risk factors for breast cancer.

## Background

Breast cancer is the most common female cancer in the U.S., with an average annual incidence rate of approximately 122 per 100,000 females [1]. In 2008, the Breast Cancer Fund published a comprehensive document reviewing the known risk factors associated with this cancer and stressed the evidence for the role estrogen plays in its development and progression [2]. This hormone and other similar compounds bind to intracellular estrogen receptors, which initiates a cascade of events that culminates in cell proliferation [3]. This process leads to an increase in breast size during puberty and pregnancy, however, unhindered it can also lead to cell mutations [3]. The use of exogenous estrogen, such as is found in oral contraceptives and hormone replacement therapies (HRT), also triggers this cell proliferation process and explains the increase risk of breast cancer for women using these products [2,4-7].

The discovery of estrogen-mimicking compounds in the environment, and the synergistic activity of many of these on estrogen receptors [8] has lead researchers to hypothesize about the role xenoestrogens (compounds in the environment that mimic estrogen) play in increasing the risk of breast cancer. Common xenoestrogens include pesticides, such as trichloromethane, chlordane, hexachlorocyclohexane, and hexachlorobenzene, industrial chemicals, such as polychlorinated biphenyls, dioxins, benzene, and polybrominated biphenyls, and vinyl chloride [2,9]. There are also some compounds such as atrazine that indirectly increase estrogen levels by activating aromatase [10]. Although the level of individuals xenoestrogens are relatively low in the environment these compounds may act synergistically [8,11]. Further, exposure to estrogenic compounds may be increasing over time as many bio-accumulate in the environment.

**Figure 1:**
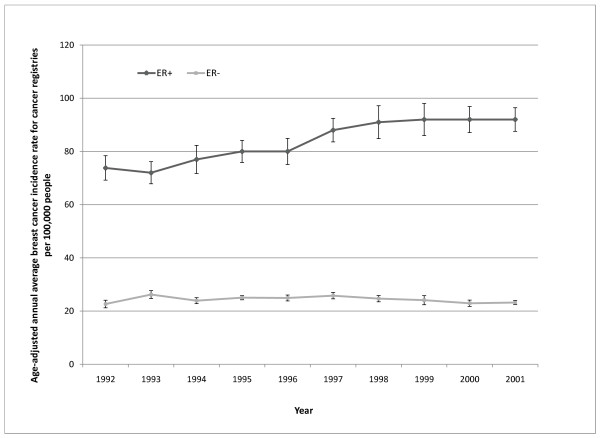
**Temporal trend between 1992 and 2001 for ER+ and ER- breast cancer rates for twelve cancer registries in the U.S**. (San Francisco- Oakland, Connecticut, Atlanta, Rural Georgia, Hawaii, Iowa, Detroit, New Mexico, Utah, Seattle, and Los Angeles). A time series analysis suggest there was a strong time effect for ER+ cancers (F_1,96 _= 168.46; p < 0.001), but not for ER- cancers (F_1,96 _= 0.92; p < 0.340). All data were obtained from the National Cancer Institute SEER program.

Although breast cancer has been associated with exposure to estrogen, not all breast cancers are responsive to this hormone and its analogs. The response of cells to estrogen depends on whether they have estrogen receptors. Classifying breast cancers by their estrogen receptor status (estrogen receptor positive (ER+) or estrogen receptor negative (ER-)) is done to assist in the selection of appropriate therapies-some ER+ cancers respond favorably to hormone blockers while ER- cancers do not. This classification scheme also provides insight into the possible pathophysiology of these tumors. Several studies have found that ER+ and ER- breast cancers have distinctly different risk factors and, therefore, possibly different etiologies [12]. In general, ER+ breast cancers are more commonly correlated with reproductive related risk factors associated with endogenous estrogen exposure, such as early menarche, number of pregnancies, and late age childbearing [12]. The different temporal pattern for age-adjusted annual Caucasian incidence rate of ER+ breast cancer compared with the rate for ER- breast cancer from 12 cancer registries between 1992 and 2001(Figure 1) also indicates these types of cancers have different risk factors. Given the different response of ER+ and ER- breast cancers to estrogen, it is possible that these disparate classifications of breast cancers also respond differently to xenoestrogens. The objectives of this study were to 1) determine whether ER+ and ER- breast cancer rates in the U. S. are associated with county level environmental factors, such as pesticide use, toxic air emissions, and pollution from urban activities, after controlling for the effects of known confounders and meteorological parameters, and 2) determine whether the association between environmental factors and breast cancer was different for ER+ and ER- breast cancers.

## Methods

### Data Collection

We extracted age-adjusted (to the 2000 U.S. standard population) average annual incidence rates (cases per 100,000 population per year) for ER+ and ER- malignant female breast cancers between 2000 and 2003 for Caucasians from the U.S. National Institutes of Health Surveillance, Epidemiology, and End Results (SEER) Program [1]. Rates for ER + and ER- were only for invasive cancers (not in situ).

Analyses were only performed on data for Caucasian females (of Hispanic and non-Hispanic origin combined) to control for the effect of race and sex. Age was controlled for by using age-adjusted rates for both cancer datasets. In total we had 439 counties from 11 states with incidence rate data for both ER+ and ER- female breast cancers. We excluded one county from our data analysis because its incidence rate of ER- breast cancer was twice that of other counties and the data could not be verified with information on the annual incidence rate of all breast cancer from the National Cancer Institute.

We acquired population demographics from the U.S. Census Bureau [13] for the counties with breast cancer data. County-level data included total population density in 2000 (number of individuals living in the county divided by the county area in square miles, used as a proxy for urban pollution), proportion of the county used to grow crops in 1997 (used as a proxy for pesticide use), and annual average county unemployment rate between 2000 and 2004 (used as a measure of the socioeconomic status). The annual average age-adjusted mortality rate from heart disease for female Caucasians between 1 and 65 years of age in 2000 and 2004 was acquired through the Centers for Disease Control and Prevention WONDER program [14].

Environmental information on average shortwave radiation, mean heating degree days (HDD- defined as the annual sum of degrees Celsius required to attain 18.3 °C when the air temperature is less than 18.3°C), mean annual vapor pressure, and mean precipitation between 1980 to1997 was obtained from DAYMET U.S. Data Center [15]. The spatial reference for these data was defined in ArcGIS (v. 9.3.1) using a projection file provided by the Utah State University Spatial Data Group [16]. Data were then re-projected to allow for the calculation of means by county using zonal statistics. Average county elevation was obtained from the National Oceanic and Atmospheric Administration's (NOAA) National Climatic Data Center (NCDC) [17]. Means per county were calculated using zonal statistics in ArcGIS (v.9.3.1).

Wind data for the U.S. were downloaded from the NOAA NCDC website [18] for years 2000-2004. Only stations with data for each of the five years of interest were included, resulting in 1193 stations spread over 941 counties across the lower 48 states. Data were available for all of the lower 48 states, with the number of stations varying between three, for small states such as Delaware and Rhode Island, to 92 for large states such as California. Average wind speeds were then interpolated for the remaining counties in ArcGIS (v. 9.3.1) using kriging with an exponential semivariogram model.

The county-level modeled ambient risk for cancer, based on the 2002 national air toxics emission inventory of known carcinogens, was downloaded from the Environmental Protection Agency (EPA) National-Scale Air Toxics Assessment program website for each county in our study [19]. The county level environmental dataset used for this study is available in Additional file 1

### Statistical analyses

We fitted an ordinary least squares regression model to both the ER+ and ER- breast cancer data using the environmental and demographic parameters in our dataset. The environmental parameters included both measurements of pollution (crop density, population density, and cancer risk associated with air emissions), as well as meteorological parameters (average wind speed, vapor pressure, shortwave radiation, HDD, and precipitation). Quadratic terms for radiation and HDD were also included, allowing for a nonlinear relationship between these variables and the response. In addition, our initial models included known confounders (a measure of socioeconomic status and premature mortality from heart disease) and biologically plausible interaction terms (Table 1).

**Table 1:** Variables included in the initial general linear models of the age-adjusted annual incidence rates of ER+ and ER- breast cancers.

	ER+			ER-		
Predictor	Coefficients	T	*p *-Value	Coefficients	T	*p *-Value
Constant	336.9	2.4	0.017	23.24	0.32	0.748
Wind speed	-0.537	-0.2	0.845	0.166	0.12	0.906
Population density	-0.0068	-0.05	0.961	0.01324	0.18	0.854
Crop density	196	0.8	0.424	230.5	1.82	0.069
Vapor pressure (VP)	-0.03189	-1.61	0.109	-0.00305	-0.3	0.766
Precipitation (Precip)	-0.0442	-0.34	0.731	-0.0851	-1.29	0.199
Radiation	-9.06	-0.57	0.568	7.177	0.88	0.381
HDD	-0.02147	-2.02	**0.044**	-0.00245	-0.45	0.656
Mortality heart disease	-0.23591	-3.54	**<0.001**	0.05616	1.64	0.103
Unemployment rate	-2.3355	-3.48	**0.001**	-0.8864	-2.56	**0.011**
EPA Cancer risk	-3943008	-0.95	0.345	-2668265	-1.24	0.215
HDD^2	1.96E-06	1.9	**0.058**	1.10E-07	0.2	0.838
RAD^2	-0.1149	-0.22	0.827	-0.3962	-1.46	0.145
Wind*EPA risk	22153	0.17	0.864	-1009	-0.02	0.988
Wind* pop density	0.002863	0.68	0.496	-0.00018	-0.08	0.935
Wind *crop density	-7.648	-1.5	0.135	-0.992	-0.38	0.707
Radiation* crop density	-6.4	-0.5	0.614	-10.008	-1.53	0.127
Radiation * pop density	-0.00114	-0.17	0.865	-0.00103	-0.3	0.765
Radiation * EPA risk	249064	1.27	0.206	155004	1.53	0.127
HDD*EPA risk	95.8	0.36	0.72	63.4	0.46	0.645
HDD* pop density	-2.60E-06	-0.36	0.716	2.00E-07	0.05	0.957
HDD* crop density	-0.00078	-0.05	0.958	-0.01216	-1.6	0.11
VP *crop density	0.00161	0.03	0.976	-0.04228	-1.56	0.12
VP * pop density	1.85E-05	0.86	0.39	7.59E-06	0.68	0.494
VP * EPA risk	-742.1	-0.98	0.326	-183.6	-0.47	0.637
Precip * EPA risk	7804	1.26	0.21	3958	1.24	0.217
Precip * crop density	-0.409	-1.17	0.243	0.0442	0.25	0.807
Precip * pop density	-6.10E-05	-0.42	0.674	-5.50E-05	-0.74	0.46

We pared down each of our initial models by successively removing the eligible variables in the model with the largest p-value. An eligible variable was an interaction term, a curvature term, or a main effect if there was no interaction or curvature term for the variable in the current model. Variables were dropped until Akaike's information criterion corrected for sample size (AICc) was minimized [20]. The predicted R^2 ^[21] and the AICc for the model that best fit ER+ breast cancers and the model that best fit the ER- breast cancer data were compared.

Variance inflation factors (VIFs) were calculated for a model with only main effects. The purpose of this model was to demonstrate the level of dependence among the meteorological variables to aid in the interpretation of their influence on ER+ breast cancer in our final model.

To clarify the relationship between ER+ breast cancer and the statistically significant interaction terms in the best fit model we graphed the relationship using the regression equation. This plot was generated by introducing the median value for all parameters except those of interest and determining the incidence of breast cancer associated with the upper and lower quartile range of values for the parameters of interest. The figures were generated in Excel (2007 Microsoft^® ^Office Excel^® ^2007).

## Results

The average annual incidence rate of estrogen responsive (ER+) breast cancer in this study was 76.9 cases per 100,000 with a range between 12.5 and 134.5 cases per 100,000 people. By comparison, the average annual incidence rate of ER- breast cancer from the same counties was 22.3 with a range between 0 and 65.0 cases per 100,000 people.

Our final model, containing several meteorological and environmental pollutant variables, explained approximately 13.5% of the variation in the county level annual incidence rate of ER- breast cancers (Table 2). By comparison, a similar model explained 38.6% of the variation in the average annual incidence of ER+ breast cancers in the same counties. The AICc of the best fit model for ER+ breast cancer rates was 195.1 points lower than the simple mean model, whereas the improvement in the AICc of the best fit model compared to the simple mean model for the ER- breast cancer rate was only 49.3 (Table 2). Differences in the AICc of 10 units or more are considered significantly large [20]. These findings suggest that the environmental parameters used in our models are more strongly associated with ER+ breast cancers than with ER- breast cancers.

**Table 2:** Summary table for the initial and final models for the rate of ER+ and ER- breast cancers including the R^2^, R^2 ^predicted, and AICc values.

	# of predictors	R^2^	R^2^- predicted	SSE	AICc*
**ER+ Models**					
Initial	29	40.90%	32.87%	116626	2512.853[172.87]
Final model	11	38.60%	35.56%	121312	2490.509[195.209]
One mean	2			197429	2685.72
					
**ER- Models**					
Initial	29	16.10%	8.40%	31017.9	1931.437[18.935]
Final model	9	13.50%	10.47%	31982	1901.04[49.33]
One mean	2			36978.3	1950.37

*[Difference in AICc between the one mean model and the specified model].

The best fit model for ER+ breast cancers included the following meteorological variables: precipitation, shortwave radiation, HDD (which is a measure of how cold a county is during the year), and mean vapor pressure (Table 3). Interpretation of the coefficients for these explanatory variables was complicated by the presence of colinearity, as evidenced by the variance inflation factors (VIF) of the main effects [21]. For example, approximately 82% of the variation in HDD was described by vapor pressure, precipitation and radiation. Colinearity made it difficult to assess the effect of each variable individually, even though the AICc and predicted R^2 ^values indicated these parameters combined were important in forming a predictive model.

**Table 3:** Variables included in the final general linear models for age-adjusted annual incidence rates of ER+ and ER- breast cancer.

Predictor	Coefficient	T	*p-*Value	VIF*	R^2 ^= 1 - 1/VIF
**ER+ final model**					
Constant	255.53	8.25	<0.001	NA	
Crop density	31.89	2.62	0.009	2.264	55.80%
Vapor pressure	-0.0415	-7.25	<0.001	5.753	82.60%
Precipitation	0.11647	2.78	0.006	4.132	75.80%
Radiation	-7.219	-5.21	<0.001	4.358	77.10%
HDD	-0.0094	-5.21	<0.001	5.477	81.70%
Mortality heart disease	-0.2766	-4.56	<0.001	1.466	31.80%
Unemployment rate	-2.0359	-3.57	<0.001	1.669	40.10%
EPA cancer risk	267850	3.26	0.001	1.424	29.80%
Precip X crop density	-0.2302	-2.01	0.045	NA	
					
**ER- final model**					
Constant	-35.86	-0.82	0.413		
Crop density	3.127	1.78	0.075		
Vapor pressure	-0.01268	-4.63	<0.001		
Radiation	13.333	2.22	0.027		
HDD	-0.00258	-2.99	0.003		
Mortality heart disease	0.05211	1.72	0.086		
Unemployment rate	-0.9082	-3.21	0.001		
RAD^2	-0.5252	-2.61	0.009		

*The variance inflation factors and the R^2 ^for each main effects variable were calculated for a model with only main effects. NA = not applicable

**Figure 2:**
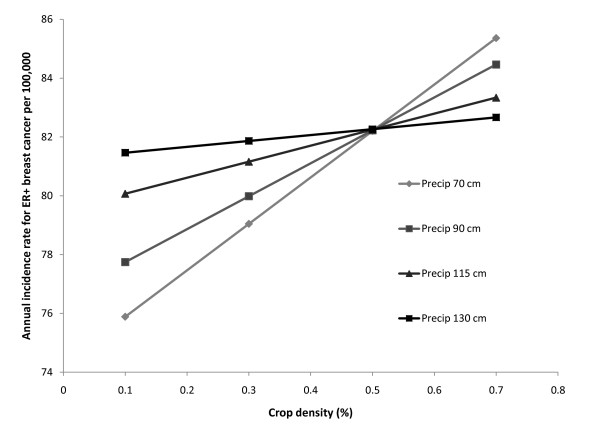
**Predicted effect of crop density (as a percentage of the county area) on the county level annual indicence rate of ER+ breast cancer between 2000 and 2003 at different levels of precipiation (in cm) using our final model and keeping all other other variables in our model constant at their median value**.

The effect of the density of crop production in the county was modified by the amount of precipitation in the county. There was a stronger positive association between the density of crops in a county and the annual incidence rate of ER+ breast cancers when there were low precipitation levels (Figure 2). The relationship between crop density and the rate of ER+ breast cancer was not as strong in counties with high annual precipitation.

Other variables that were correlated to ER+ breast cancer included EPA's calculated estimate of cancer risk based on toxic air emissions data. In general, counties with a high risk had a high rate of cancer (Table 3 and Figure 2). Both demographic variables, premature mortality from heart disease and unemployment rate, were negatively associated with ER+ breast cancer rates (Table 3). As these variables increased the rate of cancer decreased.

## Discussion

Our final model, with several environmental variables, explained approximately 38% of the variation in the age-adjusted average annual incidence rate of ER+ breast cancer for Caucasian women. In contrast, we were only able to explain approximately 14% of the variation in the age-adjusted annual incidence rate of ER- breast cancer with the same set of environmental variables (Table 2). The difference between our final models for these two classifications of breast cancers suggests that in the same counties ER+ and ER- breast cancers have different risk factors and that environmental factors play a greater role in explaining ER+ cancers than ER- cancers.

Two of the three measures of environmental pollution, the EPA cancer risk estimate derived from 2002 toxic air emissions and the proportion of land in a county used to grow crops, a proxy for pesticide use, were positively associated with ER+ breast cancers (Table 3). Interestingly, in the same counties, ER- breast cancer rates were not significantly associated with either of these two pollution indices (Table 3), which suggests that different factors are driving the rate of breast cancers without estrogen receptors. Both of the pollution variables associated with ER+ breast cancer rates measure exposure to a range of different pollutants in a county, so it was not possible to identify which of the chemicals captured within these variables was most associated with breast cancer. Several of the chemicals included in these aggregated measures of pollution are known endocrine disruptors. For example, at least 8 of the 86 chemicals used to create the EPA cancer risk measurement were xenoestrogens [2,9] and 27 others are on the EPA's Tier 1 list of chemicals for screening [22]. Further, some pesticides/herbicides, including the commonly used compound atrazine, have effects on estrogen sensitive tissues [10,23]. It is therefore possible given the differences in their response to estrogen that the difference in the association between the pollution indices and ER+ and ER- breast cancers is due to estrogen mimicking compounds found in the environment. Further research is required to confirm this hypothesis and elucidate the specific chemicals associated with ER+ breast cancer.

We included meteorological variables in our model to control for confounding between climatic variables, pollution, and breast cancer. It has been shown by several other researchers that exposure to ultraviolet (UV) radiation, which increases levels of Vitamin D in individuals, reduces the risk of breast cancer [24]. Because exposures to UV radiation and pollutants are both highly dependent on geographic location we wanted to control for confounding between radiation and pollutants. Further, meteorological variables, such as radiation, temperature, and relative humidity have a direct effect on the deposition, degradation, and adsorption of organic pollutants [25-29]. Including temperature, precipitation, and water vapor pressure, as well as their interaction terms, in our model adjusted for the effects these factors have on organic pollutants. All of these variables were associated with ER+ breast cancer in our final model (Table 3), but it was difficult to decipher the precise role of individual variables on cancer because they were highly correlated (VIFs in Table 3). A dataset with more than 439 counties would improve our understanding of the relationship between specific meteorological variables and breast cancer. We chose to include all the variables in our final model because all were significant, and the models with these variables had the best AICc and predicted R^2^; however, the high level of correlation between meteorological variables, especially between temperature and vapor pressure, made it difficult to interpret these individually.

There were a few variables in our model that we expected to be significantly associated with breast cancer rates that were not. For example, we did not find an association with population density (Table 1 and 3), as has recently been reported by Crouse et al. [30]. This may be because the air emissions from urban activity associated with breast cancer were partially captured in the EPA's measure of cancer risk, which was based on 86 chemicals. We found a positive association between the EPA's measure of cancer risk and ER+ breast cancer. It may also be that population density (our measure of urban pollution) was not sufficiently refined to detect the association between urban air pollution and breast cancer that was detected in the study by Crouse et al. [30].

A limitation of this study was the fact that we could not include several known risk factors for breast cancer because the data were not available at a county level for the 11 states included in this study. Factors such as exogenous hormone use (e.g. oral contraceptives and HRT), smoking, ethnicity, and obesity may cluster spatially and are associated with breast cancer [2]; thus, they have the potential to distort the associations between pollutants, meteorological parameters, and the incidence of breast cancer. Although we could not control for these potential confounders directly we may have indirectly controlled for the effects of ethnicity, cancer detection method, use of contraceptives and HRT, smoking, and obesity by controlling for other parameters such as race, unemployment rates, and premature mortality from heart disease. By only including Caucasian women in our analyses we removed the effect of race, but our findings are limited to this group of females. Preliminary data analysis of ER+ breast cancer rates in African American women suggests these cancers are not as strongly correlated to environmental pollutants as the ER+ breast cancers of Caucasian women (Data not shown). Further research is required to better understand the differences between types of breast cancers and race.

Premature mortality is correlated with smoking and obesity so by including this variable in our model we likely controlled, partially at least, for smoking and obesity at the population level. Unemployment rate is correlated with socioeconomic status (SES) and education, both of which are correlated with oral contraceptive use [31,32]. The correlation between the use of oral contraceptives and SES may explain the negative association between unemployment rate and breast cancer observed in this study and by others [33].

Since ER+ breast cancers are more commonly found in older women [34] and screening mammograms are more frequently used in older women, the detection method may have resulted in a greater relative increase in the age-adjusted rates for ER+ cancers than for ER- cancers. If mammogram screening was positively correlated with environmental pollutants this could partially account for the correlation observed between environmental pollutants and ER+ breast cancers. Although this type of bias is possible we did not find a positive correlation between unemployment rate (a surrogate for SES and mammogram use) and crop density and the EPA cancer risk score when we evaluated the variables individually (Additional file 1). This potential type of confounding is difficult to control for in an ecological study, especially when we only have surrogate variables for the parameters of interest, so the results of our study should be interpreted with caution.

There are several other limitations that are common to most ecological studies and that limit the conclusions that can be derived from this type of study. For example, misclassification due to migration likely occurred in our study, given the long latency period of breast cancer. If we assume that migration occurred in all directions, it would have biased our results towards the null reducing our ability to detect a significant association. Misclassification of the ER status of cancers may also have occurred; again, this error most likely biased our results towards the null. Also limiting our interpretation of the associations found in this study is the fact that all measurements were aggregated at the county level, so we cannot conclude that associations between environmental risk factors associated with the rate of breast cancer in a county apply to the individual. For this reason, we can only hypothesize that the different environmental variables found to be associated with the rate of ER+ breast cancer at the county level may also transfer to the individual risk and should be further investigated. Despite these limitations, the models appear to be biologically plausible, and at least two of the significant relationships with breast cancer noted in this study, short wave radiation and SES, have been validated by others [2,33,35].

## Conclusions

This study, therefore, provides evidence at a county level that breast cancer rates, specifically ER+ breast cancer rates, are correlated with environmental factors, including broad categories of pollutants that are known to include endocrine disruptors. The higher the EPA cancer risk estimate and the greater the proportion of land used to grow crops, especially in dry climates, the higher the rate of ER+ breast cancer. This relationship was not apparent with the ER- breast cancer rates in the same counties, which lends further credence to the hypothesis that breast cancers that are sensitive to estrogens may be influenced by environmental endocrine disruptors. The significance of this study was twofold. First, it identified groups of environmental pollutants that were associated with county level ER+ breast cancer rates and second, it suggest that ER+ and ER- breast cancer have different risk factors, and therefore, should be separated in future observational studies that seek to identify risk factors for breast cancer.

## Abbreviations

AICc: Akaike's information criterion corrected for sample size; CDC: Centers for Disease Control and Prevention; EPA: Environmental Protection Agency; ER+: estrogen receptor positive; ER-: estrogen receptor negative; HDD: heating degree days which is the annual sum of degrees Celsius required to attain 18.3 °C when the air temperature is less than 18.3°C; HRT: hormone replacement therapy; NCDC: National Climatic Data Center; NOAA: National Oceanic and Atmospheric Administration; SEER: Surveillance, Epidemiology, and End Results; SES: Socioeconomic status.

## Competing interests

The authors declare that they have no competing interests.

## Authors' contributions

SS provided the idea for the project, assisted with the data interpretation, and helped write the manuscript. RM developed the hypothesis for the project, extracted the cancer and health data, and helped with the analysis of the data and writing of the manuscript. SM and AC extracted and geo-referenced all the environmental data. DD conducted all the statistical analyses and helped with the interpretation of the data. All authors participated in the review and final approval of the manuscript.

## Supplementary Material

Additional file 1 **Environmental dataset used for our analyses**. Sheet one of the excel file contains the data and sheet two contains a description of the variables. Data on breast cancer rates are available through SEER http://www.seer.cancer.gov. Click here for file
